# Clinicohematological and cytogenetic profile of myelodysplastic syndromes in Pakistan-compare and contrast

**DOI:** 10.1186/s13039-017-0318-4

**Published:** 2017-05-08

**Authors:** Nida Anwar, Aisha Arshad, Muhammad Nadeem, Sana Khurram, Naveena Fatima, Sumaira Sharif, Saira Shan, Tahir Shamsi

**Affiliations:** National Institute of Blood Disease and Bone Marrow Transplantation (NIBD), St 2/A block 17 Gulshan-e-Iqbal KDA scheme 24, Karachi, Pakistan

**Keywords:** Myelodysplastic syndromes, Clinicohematological characteristics, Cytogenetics, Karyotype

## Abstract

**Background:**

Myelodysplastic syndromes (MDS) are clonal stem cell disorders exhibiting cytopenias, ineffective hematopoiesis and morphological dysplasia. Bone marrow cytogenetics, inspite of being incorporated as mandatory tool in diagnosis are done less frequently due to limited availability of this technique in Pakistan. The aim of the study was to study baseline clinicohematological and cytogenetic characteristics of patients presenting with de novo MDS.

**Results:**

A retrospective cross sectional study was done at National Institute of Blood Diseases and Bone Marrow Transplantation, Karachi, Pakistan from 2010 to 2016. Total of 177 patients were included in the study having median age 51 years and male to female ratio of 3:1. Pancytopenia was observed in 80 (45%) patients and bicytopenia in 74 (42%). Mean Hb% was 7.8 ± 2.18 g/dl, total leukocyte count (TLC) 8.8 ± 13.6 × 10^9^/l, platelet count was 82 ± 95.7 × 10^9^/l. Of total 170 (96%) were transfusion dependent. Refractory cytopenias with multilineage dysplasia (RCMD) was the most common world health organization (WHO) category. Karyotype was done in 98 (55%) patients out of which 44 (45%) had abnormal karyotype, complex karyotype (CK) was most commonly observed in 12 (12.2%) followed by monosomy 7 in 7 (7.1%).

**Conclusions:**

We found younger median age at diagnosis, higher mean TLC and no significant history of recurrent infections. CK and monosomy 7 carry bad prognostic implications and early disease transformation to acute myeloid leukemia (AML). Monosomy 7 being associated with bad overall survival, such patients must be identified early with close clinical follow up and offered stem cell transplant. This is the largest cohort of patients of MDS evaluated for baseline clinical and cytogenetic characteristics in our country.

## Background

Myelodysplastic syndromes (MDS) are group of clonal hematopoietic stem cell disorders exhibiting ineffective hematopoiesis, morphological dysplasia and progressive tendency to evolve into acute myeloid leukemia (AML) [1–5]. The exact pathogenesis is not completely understood [3]. However, proposed pathogenic causes include increased apoptosis, immunological abnormalities along with clonal basis [5]. The disease can be classified into primary (de novo) and secondary MDS, whether it is de novo or arise as result of previous radiochemical exposure [1]. Consensus International Prognostic Scoring System (IPSS) is used for predicting outcome and planning therapy in MDS which includes number of cytopenias, percentage of bone marrow blast and cytogenetics [5]. Thus the role of cytogenetics with respect to diagnosis and prognosis has been well established in this clonal disorder [5]. However in developing countries like Pakistan, with poor socioeconomic status of patients, clinicians have very limited availability of sophisticated techniques like cytogenetics inspite of this being incorporated as mandatory tool for the diagnosis [5]. Thus most of the cases of refractory cytopenias are not diagnosed for MDS. Impact of racial difference on disease biology and clinical behavior was evaluated in previous Asian study but has not been well established [6]. Keeping this in mind, this study was done to assess the baseline clinicohematological characteristics of patients presenting with MDS, evaluate their cytogenetic profile and compare our analysis to what has been reported previously. This is the largest cohort of patients diagnosed with MDS and evaluated for their baseline hematological, clinical and cytogenetic profile in our country.

## Methods

This retrospective cross sectional study was conducted at National Institute of Blood Diseases and Bone Marrow Transplantation, Karachi Pakistan from June 2010 to June 2016. Baseline investigations done included complete blood counts, serum vitamin B 12, serum and RBC folate levels. Clinical parameters were recorded. Bone marrow biopsy samples were taken from posterior superior iliac spine through jamshidi needle and were stained by leishman’s stain. Perl’s (Iron) stain was carried out on each bone marrow sample by commercially provided kits from merck diagnostic according to manufacturer’s instructions. Cytogenetic analysis was performed on overnight, 24-h un-stimulated and 72-h stimulated bone marrow cultures using standard procedures. The GTG (G-bands via trypsin using Giemsa) banding technique was applied, karyotypes were described according to the International System for Human Cytogenetic Nomenclature (ISCN) 2013, karyogram were made using Meta system. Patients were classified according to world health organization (WHO) 2008 classification and IPSS was also calculated. Approval from the Institutional ethics committee was obtained prior to the study.

### Inclusion criteria

During the study period, patients diagnosed as de novo MDS based on morphological and/or cytogenetic basis were included in the study. Thorough morphological assessment of peripheral smears and bone marrow biopsy was done along with bone marrow cytogenetic analysis.

### Exclusion criteria

Patients presenting with cytopenias and normocytic/macrocytic anemia due to other non malignant causes, patients having history of prior chemotherapy or irradiation and patients having organomegaly or lymphadenopathy were excluded from the study.

### Statistical analysis

Statistical analysis was done by statistical package for the social sciences version 22.0 (SPSS Inc, Chicago, IL, USA). Descriptive variables were calculated as mean, standard deviation (SD), frequencies and percentages.

## Results

**Fig. 1:**
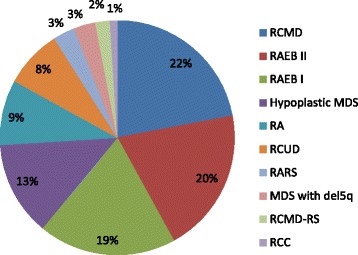
Frequency of MDS patients according to WHO subtype. RCMD = Refractory Cytopenia(s) with Multilineage Dysplasia. RAEB II = Refractory Anemia with Excess Blasts II. RAEB I = Refractory Anemia with Excess Blast I. RA = Refractory Anemia, RCUD = Refractory Cytopenias with Unilineage Dysplasia, RARS = Refractory Anemia with Ringed Sideroblasts, RCMD-RS = Refractory Cytopenias with Multilineage Dysplasia and Ringed Sideroblasts, RCC = Refractory Cytopenias of Childhood

**Fig. 2:**
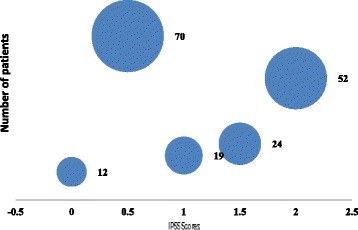
IPSS scoring of patients

A total of 177 consecutive patients diagnosed with MDS were included in the study. The median age of patient was 51 (range 3 to 90 years). The male to female ratio was 3:1. Frequency of all patients according to WHO classification 2008 is shown in (Fig. 1). IPSS scoring of patients is given in (Fig. 2). Mean hemoglobin (Hb), total leukocyte count (TLC), platelets, MCV at baseline and subtypes of MDS as per WHO classification in comparison with other national and international studies is shown in Table 1 [1–3, 6–15].

**Table 1:** Hematological characteristics in comparison with national and international studies

Variables	Present study	Pakistan ^1^	Pakistan ^2^	Pakistan ^3^	China ^6^	Korea ^7^	India ^8^	Turkey ^9^	Greece ^10^	Taiwan ^11^	Singapore ^12^	Spain ^13^	Germany ^14^	Poland ^15^
Total No. of Patients	177	45	46	71	508	176	40	54	855	189	43	640	216	863
Median age(years)	51.7	64	Mean 43.09	60	49	57	42	67	73.1	56	Mean 64	66	73	70
Mean Hb (g/dl)	7.8	7.7	6.52	–	6.3	–	6.84	9.0	9.5	8 .0	7.7	–	–	9.1
Mean MCV (fl)	90	89.4	89.4	–	–	–	–	–	–	–	–	–	–	97
Mean TLC (×10^9^/l)	8.8	5.7	3.4	–	3.0	–	4.45	3.8	7.52	3.3	5.6	–	–	–
Mean Platelet (×10^9^/l)	82	83	60	–	42	–	85	163	158	61	101	–	–	129
MDS Category														
RCMD %	22	53.3	52.2	52	–	37	10	–	14.3	–	–	-	31	28.3
RAEB-II %	19.7	4.4	6.5	23.9	21.1	21	15	30	14.4	18.5	7	12	11	19.6
RAEB-I %	19.2	4.4	6.5	11.3	27	29	18	7	18.9	40.7	16.3	32	8	13.9
Hypoplastic MDS %	13	–	–	–	–	–	–	–	–	–	–	–	–	–
RA %	9	–	–	8.5	43.9	12	37	57	20.6	32.5	38.1	29	9	19.7
RARS %	7.9	–	–	2.8	2.8	–	7.5	–	6.2	8	14	12	4	6.7
RCUD %	3.3	22.2	RCUD–RA 26	–	–	–	–	–	–	–	–	–	–	–
RCMD-RS %	2.8	–	2.2	1.4	–	–	2.5	–	2.8	–	–	–	13	2.1
MDS with 5q %	2.20	–	–	–	–	–	10	6	1.9	–	–	–	2	4.6
RCC %	0.5	–	–	–	–	–	–	–	–	–	–	–	–	–
MDS-U %	–	15.5	6.5	–	–	1	–	–	–	–	–	–	–	5.1

Comparison of cytogenetic profile of our patients with other national and international data is shown in Table 2 [1–3, 6–15].

**Table 2:** Cytogenetic profile in comparison with national and international studies

Variables	Present study	Pakistan ^1^	Pakistan ^2^	Pakistan ^3^	China ^6^	Korea ^7^	India ^8^	Turkey ^9^	Greece ^10^	Taiwan ^11^	Singapore ^12^	Spain ^13^	Germany ^14^	Poland ^15^
Cytogenetics available/Total No. of patients	98/177	–	–	71/71	367/508	–	40/40	54/54	591/855	175/189	40/43	640/640	–	276/863
Normal Karyotype %	55	–	–	57.7	62.9	–	53	85	61.6	53.1	52.5	49	–	44.6
Abnormal Karyotype %	44	–	–	42.3	37.1	–	47	15	38.4	46.8	47.5	51	–	55.4
Complex Karyotype %	12	–	–	15.5	38.9	–	–	–	7.6	17.2	20	14	–	6.4
Monosomy 7%	7.1	–	–	–	–	–	32						–	–
Del(5q) %	6.1	–	–	2.8	12.5	–		6	2.7	9.1	15	5	–	19
Del(7q) %	2	–	–	4.2	4.4	–			3	14.9	7.5	4	–	1.8
Trisomy 8%	3	–	–	9.9	25.7	–	16	9	8.3	12	10	5	–	2.2
Del(20q) %	4	–	–	1.4	14.7	–			2.2	6.3		1	–	0.3
Trisomy 21%	1	–	–	–	–	–	–	–	–	–	–	–	–	–
t(1;9) (q11;q34) along with Del(9q)^a^ %	1	–	–	–	–	–	–	–	–	–	–	–	–	–
Trisomy 8 with Del(7q)^b^ %	1	–	–	–	–	–	–	–	–	–	–	–	–	–
t(6:9) (p23;q23)^b^ %	1	–	–	–	–	–	–	–	–	–	–	–	–	–
Monosomy 7,8^b^ %	1	–	–	–	–	–	–	–	–	–	–	–	–	–
Del(9q)^b^ %	2	–	–	–	–	–	–	–	–	–	–	–	–	–

^a^unique cytogenetic findings in our study

^b^rare cytogenetics

Moreover, we observed the cytogenetics of our patients in each WHO category which is summarized in Table 3.

**Table 3:** Cytogenetics of patients in each WHO category

	Total No. of cytogenetic performed *N* = 98	Normal karyotype *N* = 54	Abnormal karyotype *N* = 44	Chromosomal Abnormalities (N)
WHO subtype:	(%)	(%)	(%)	
RCMD	22.4	10.2	12.2	t(6;9) (p23;q23) (1) Del(20q) (2) + 8 (1) −7 (4) 46,XY,+3,del(5q),del(10q) (1) 48,XY,del(5q),+8 + 11,del (20q) (1) 47,XY,del(5q),+8,del(20q) (1) 49,XY,del(5q),del(7q),+8 + 11 + 17,del(20q) (1)
RAEB II	27.5	13.2	14.20	Del(9q) (1),del(5q) (1), del(7q) (2), del(20q) (2) + 8 (1) −7 (1),−8 (1),−11 (1) 45,XY,t(6;9) (P23;q34),−3,+17 (1) 46,XY,t(1;9), del(9q),+11 (1) 47,XY,del(5q), +8 del(20q) (1) 49,XY,del(5q),del(7q),+8,del(11q),+17,del(20q) (1)
RAEB-I	16.32	7.14	9.1	Del(9q) (1), Del(20q) (1) +8 (1) −7 (2),−7,8 (1) 45,XY,del(5q),del(7q),-Y (1) 47,XX,t(3;12) (q26.2;q22),del(7q),+15 (1) 47,XY,+11 + 17,−22 (1)
Hypoplastic MDS	11.2	9.1	2.04	+8 with del(7q) (1) +12 (1)
RA	7.14	6.12	1.02	47,XY,+11,+17,del(20q) (1)
RCUD	5.1	4.08	1.02	t(1;9) (q11;q34) along with del(9q) (1)
RARS	2.04	2.04		–
RCMD-RS	2.04	2.04		–
MDS with 5q	5.10		5.10	Del5q (5)
RCC	1.02	1.02		–

In our patients the most common presenting complaint was loss of appetite in 173 (98%) followed by weakness 141 (80%) and fever 83 (47%). Absolute neutophil count (ANC) of <1.8 was found in 78 (44%) and >1.8 was found in 99 (56%) of patients. Pancytopenia was observed in 80 (45%) and bicytopenia in 74 (42%) (anemia and thrombocytopenia). However, 23 (13%) had cytopenia of one cell lineage. One hundred and seventy (96%) patients were transfusion dependent. History of recurrent infection was found in 21 (12%). Bacterial infections were observed to be the most common cause followed by viral and fungal infections. Co morbidities were observed in 116 (66%) of patients including hypertension in 61 (35%), diabetes mellitus in 55 (31%) while 61 (34%) had no known co morbid. Iron grading was done on all the bone marrow aspirate samples by Perl’s staining method. Grade III iron was found to be frequent, seen in 57 (32%) followed by grade IV in 38 (21.5%) and grade I in 36 (20.3%). Bone marrow myelofibrosis (MF) was done according to WHO 2008 myelofibrosis grading system and found to be MF -0 in 77 (44%), MF -I in 70 (39.5%) and MF-II in 30 (16.9%). Out of all patients, 97 (55%) were alive, 80 (45%) had died by the end of study period. The cause of death for 30 (38%) patients could not be ascertained whereas in rest of the patients the causes of death were septicemia in 19 (23.7%), severe anemia in 10 (12.5%), cardiac arrest in 15 (18.7%) and intracranial bleeding in 6 (7.5%) patients.

## Discussion

Myelodysplastic syndromes are characterized by ineffective hematopoiesis with blood cytopenias and morphological dysplasia [1–5]. MDS is generally considered a preleukemic disorder and is prevalent in elder individuals in western world [5]. However, median age at diagnosis in our study was younger which concurs with other studies done in Asia [1–4, 6–8] as compared to the studies from west with higher median age in MDS (Greece, Germany, Poland) [10, 14, 15]. In our study we observed male predominance which is universally seen in this disorder [1–8]. However, male to female ratio was 3:1 which is slightly higher as compared to previous studies [1–4, 6, 7] and somewhat comparable to what has been observed in Greece [10]. The possible explanation of gender in our region could be low literacy rate in our country and male predominant society where females seek medical attention less frequently. The most common presenting complaint in our patients was loss of appetite followed by weakness. In our study mean Hb was 7.7 g/dl which is in concordance with a previous national study [1] and higher as compared to other studies done in Pakistan, China and India [2, 6, 8] and lower as compared to Turkey, Greece and Poland [9, 10, 15]. In our study, mean platelet count was 82 ×10^9^/l, which is in concordance with a national study and also seen in India [1, 8]. Interestingly data from Turkey and Greece reveals normal platelet count in MDS [9, 10]. Mean TLC count of 8.8 ×10^9^/l in our study was higher as compared to other regional and international studies (Table 1) [1, 2, 6, 8, 9, 11, 12].

Anemia was most common presentation in previously reported studies and same was observed in our patients [1, 2]. History of recurrent infections is seen in MDS [5]. However, only 11% of our patients had history of recurrent infections, bacterial infections being most common. In our study 80 (45%) of patients presented with pancytopenia and 74 (42%) with bicytopenia having anemia and thrombocytopenia. Similar trend was observed by previous study done on regional level [1].

In our study refractory cytopenia with multilineage dysplasia (RCMD) was the most common encountered WHO sub category of MDS followed by refractory anemia with excess blast (RAEB) as observed in previous study from Pakistan [3] and the least common WHO sub category was refractory cytopenia with unilineage dysplasia (RCUD) which however contrasts with other national studies [1, 2]. On the other hand refractory anemia (RA) was most commonly observed in China and Greece [6, 10]. In our study, hypoplastic MDS was seen in 23 (13%) which is an interesting finding not observed in previous studies [1–3, 6–15]. Refractory cytopenia of childhood (RCC) was observed in only one of our patients. However we compared the classification with some previous studies in which they have followed French American British (FAB) classification [6, 7, 11–13] which is one of our study limitations.

In this study we found abnormal karyotype in 44 (45%), complex karyotype (CK) being most common in 12 (12.2%) followed by monosomy 7 in 7 (7.1%). Monosomy 7 is not commonly observed in previous studies [3, 6, 9–13, 15] except for one study from India showing higher frequency [8]. CK in our study was observed to be lower as compared to China [6]. Trisomy 8 was seen in 3 (3%), lower as compared to other studies [3, 6, 8, 10, 11]. Patients with RAEB II revealed higher frequency of chromosomal abnormalities, comparable to previously reported data [3]. In one of our patients isolated del(9q) was seen and in other del(9q) was seen along with t(1;9) (q11;q34). Del(9q) is associated with AML but very rarely reported in MDS [16] and to the best of our knowledge and searched literature t(1;9) has not been reported in MDS before. Isolated del(5q) was seen in one of our female patient with RAEB II who presented with pancytopenia which is in contrast to the clinical presentation of MDS with del(5q) syndrome [17].

Cytogenetic studies are done at limited centers in Pakistan, our study could be helpful to outline cytogenetic characteristics of MDS in our region. Karyotypic abnormalities exhibit a significant role in diagnosis and prognosis of MDS. We observed CK and monosomy 7 frequent in our study which carries poor overall survival and early transformation to AML. Such patients must be identified early in the disease course. Since our study was retrospective, future prospective studies are needed to ascertain the findings.

## Conclusion

In our study we observed younger median age of disease presentation, higher mean TLC count and no significant history of recurrent infections. RCMD was the most common WHO category and CK was most common abnormal karyotype followed by monosomy 7. Since both carry adverse prognostic implications, early identification of such patients with close clinical follow up and upfront allogenic stem cell transplant must be considered keeping in view the younger age in our cohort at time of presentation. This study was done retrospectively yet represents a large cohort of MDS in our country. In future, prospective studies are needed to be done to further elaborate disease biology and clinical outcome of the baseline adverse disease characteristics observed in our study. Also molecular testing in MDS must be incorporated since nowadays diagnostic spectrum of MDS is moving rapidly towards molecular analysis and its relation to disease outcome.

## Abbreviations

AML: Acute myeloid leukemia
ANC: Absolute neutophil count
GTG: G-bands via trypsin using Giemsa
Hb: Hemoglobin
IPSS: International Prognostic Scoring System
ISCN: International system for human cytogenetic nomenclature
MDS: Myelodysplasticsyndromes
RA: Refractory anemia
RAEB I: Refractory anemia with excess blast I
RAEB II: Refractory anemia with excess blasts II
RARS: Refractory anemia with ringed sideroblasts
RCC: Refractory cytopenia of childhood
RCMD: Refractory cytopenia(s) with multilineage dysplasia
RCMD-RS: Refractory cytopenias with multilineage dysplasia and ringed sideroblasts
RCUD: Refractory cytopenias with unilineage dysplasia
TLC: Total leukocyte count
WHO: World Health Organization

## References

[1] Sultan S, Irfan SM (2016). Adult primary myelodysplastic syndrome: experience from a tertiary care center in Pakistan. APJCP.

[2] Ehsan A, Aziz M (2010). Clinico-haematological characteristics in pakistani patients of primary myelodysplastic syndrome according to World Health Organization classification. JCPSP.

[3] Rashid A, Khurshid M, Shaikh U, Adil S (2014). Chromosomal abnormalities in primary myelodysplastic syndrome. JCPSP.

[4] Sultan S, Irfan SM, Jawed SN (2016). Spectrum of the WHO CLassification *De Novo* myelodysplastic syndrome: experience from Southern Pakistan. APJCP.

[5] Malcovati L, Hellström-Lindberg E, Bowen D, Adès L, Cermak J, Del Cañizo C (2013). Diagnosis and treatment of primary myelodysplastic syndromes in adults: recommendations from the European LeukemiaNet. Blood.

[6] Chen B, Zhao WL, Jin J, Xue YQ, Cheng X, Chen XT (2005). Clinical and cytogenetic features of 508 Chinese patients with myelodysplastic syndrome and comparison with those in Western countries. Leukemia.

[7] Lee JH, Shin YR, Lee JS, Kim WK, Chi HS, Park CJ (2003). Application of different prognostic scoring systems and comparison of the FAB and WHO classifications in Korean patients with myelodysplastic syndrome. Leukemia.

[8] Chaubey R, Sazawal S, Dada R, Mahapatra M, Saxena R (2011). Cytogenetic profile of Indian patients with de novo myelodysplastic syndromes. Ind J Med Res.

[9] Demirkan F, Alacacioglu I, Piskin O, Ozsan HG, Akinci B, Ozcan AM (2007). The clinical, haematological and morphological profile of patients with myelodysplastic syndromes: a single institution experience from Turkey. Leuk Lymph.

[10] Avgerinou C (2013). The incidence of myelodysplastic syndromes in Western Greece is increasing. Ann Hematol.

[11] Huang T-C (2008). Comparison of hypoplasticmyelodysplastic syndrome (MDS) with normo-/hypercellular MDS by international prognostic scoring system, cytogenetic and genetic studies. Leukemia.

[12] Lau LG (2004). Clinico-pathological analysis of Myelodysplastic syndromes according to French-American-British Classification and International Prognostic Scoring System. Ann Acad Med Singapore.

[13] Sole F, Espinet B, Sanz GF, Cervera J, Calasanz MJ, Luno (2000). Incidence, characterization and prognostic significance of chromosomal abnormalities in 640 patients with primary myelodysplastic syndromes. Br J Haematol.

[14] Neukirchen J, Schoonen WM, Strupp C, Gattermann N, Aul C, Haas R (2011). Incidence and prevalence of myelodysplastic syndromes: data from the düsseldorf MDS-registry. Leuk Res.

[15] Mądry K, Machowicz R, Waszczuk-Gajda A, Drozd-Sokołowska J, Hołowiecka BS, Wiater E (2015). Demographic, hematologic, and clinical feature of myelodysplastic syndrome patients: results from the first polish myelodysplastic syndrome registry. Acta Haematol.

[16] Vigue F (1998). del(9q) solely. Atlas Genet Cytogenet Oncol Haematol.

[17] Boultwood J, Fidler C, Strickson AJ, Watkins F, Gama S, Kearney L (2002). Narrowing and genomic annotation of the commonly deleted region of the 5q- syndrome. Blood.

